# Awareness of and Attitude to Breast Self-Examination and Breast Cancer Among Females in Jeddah, Saudi Arabia

**DOI:** 10.7759/cureus.36595

**Published:** 2023-03-23

**Authors:** Gutaybah S Alqarni, Mohammed T Musslem, Rayan M Alosaimi, Feras F Filfilan, Ali S Al Qarni, Hisham Rizk

**Affiliations:** 1 Faculty of Medicine, University of Jeddah, Jeddah, SAU; 2 Obstetrics and Gynecology, Prince Sultan Military Medical City, Riyadh, SAU; 3 General Surgery, Faculty of Medicine, University of Jeddah, Jeddah, SAU

**Keywords:** saudi arabia, jeddah, examination, self, cancer, attitude, awareness

## Abstract

Background

Breast cancer is the most common of all female cancers worldwide. A large percentage are diagnosed at a late stage, which can be related to awareness and knowledge deficiency. We aimed to assess the level of knowledge of and attitude to breast cancer and breast self-examination in Jeddah, Saudi Arabia.

Methodology

A descriptive cross-sectional study was carried out among 392 women in Jeddah, Saudi Arabia. Using a non-probability sampling technique, a self-administered validated questionnaire was distributed via social media. Inclusion criteria were ages above 18 years old of all educational levels.

Results

Out of 392 participants, there were 146 in the age group of 19-25 (37.2%). Most of the participants are aware of breast cancer (94.9%). The mean knowledge score was 6.9 ± 3.36. Ninety-two percent (92%) of participants had poor knowledge. Most respondents reported that the main risk factor for breast cancer was family history (83.7%). About 37% believed that the purpose of the breast self-examination practice is advice from a health care professional followed by routine examination (37.3%). About 97% agreed that early detection of breast cancer increases the chance of recovery.

Conclusion

There is a lack of knowledge and awareness about the risk factors and symptoms of breast cancer. Despite a positive attitude toward breast self-examination, it is poorly practiced.

## Introduction

Breast cancer became the most common of all cancers around the world with approximately 2.3 million new cases in 2020, overtaking most of the cancer percentages, over lung cancer. It has become the reason for 685,000 deaths worldwide [1,2].

In Saudi Arabia (SA), the number of new cases and the chance of mortality for breast cancer has been increasing over the years. By 2030, the number of new cases is expected to reach 3549. In 2016, the cases reported reached 3400. In addition, 50% of cases are diagnosed at a late stage as compared to 20% in developed countries [3-5].

Several causes, including the decreased level of awareness, poor education, wrong beliefs, and cultural barriers, may justify the high percentage of late diagnoses and deaths of breast cancer [6,7]. Mammograms, clinical breast examinations, and breast self-examination (BSE) are the standard screening methods available. Besides the debates about the efficacy of the latter, it is believed to be a useful tool in conservative societies such as SA [8,9].

The level of knowledge and awareness among females regarding breast cancer along with the attitudes toward screening methods, including the performance of BSE and being familiar with how their breasts should normally look and feel, are crucial factors in the process of early detection of breast cancer, which is essential in reducing breast cancer-related morbidity and mortality, as treatment is highly effective in the early stages of the disease [1,8,10]. A study among 508 Saudi women evaluated the attitudes and the level of awareness of BSE and breast cancer, it showed a good level of breast cancer awareness but on the other hand, BSE practice was not adequate [10]. Moreover, in 2019, a study of 422 university students assessed the level of knowledge, attitude, and practice of BSE toward breast cancer and found that they have adequate knowledge of BSE and a good attitude toward BSE. However, most of their sample does not practice BSE [11]. Another study indicates that the participants had a good knowledge of both breast cancer and BSE. But, most of them don’t practice BSE [12]. Lastly, a study conducted among 506 female undergraduate students demonstrated an acceptable level of knowledge [13].

Due to late detection and rapid morbidity growth in previous years, it raises the future concern of breast cancer, therefore, we aim to assess the level of knowledge of and attitude toward breast cancer and BSE among females in Jeddah, Saudi Arabia.

## Materials and methods

Study group

We conducted a descriptive cross-sectional study from April to September 2022. Our study sample included all females of any educational level and aged above 18 years old in Jeddah. The sample size was estimated by using the Raosoft sample size calculator [14]. Considering a 95% confidence level, a margin of error of 5%, and a response distribution of 50%, the minimal sample size required is 385. The present study included 392 participants.

Questionnaire

We used a previously validated and published questionnaire with permission [12]. The questionnaire has two parts. The first part consisted of five demographic questions, including age, marital status, nationality, educational level, occupation, and one question about whether the participant has a first-degree family history of breast cancer or not. The second part consisted of questions about (1) awareness of breast cancer, (2) knowledge of breast cancer, including risk factors and symptoms, (3) knowledge of breast self-examination, and (4) attitude toward BSE. Each question on breast cancer knowledge was given a score of one (18 questions in total). Participants were categorized into good and poor knowledge groups. Participants who scored > 60% are considered to have good knowledge and those who scored ≤ 60% are considered to have poor knowledge.

Data collection, processing, and analysis

The online self-administered questionnaire was distributed to the female population via social media, using a snowball non-probability sampling technique, and participants were encouraged to share it. Data entry was done by using Microsoft Office Excel 2016 (Microsoft Corporation, Redmond, WA). Categorical data are described in numbers and percentages using tables and figures while continuous data are presented as means and stander deviation. A one-way analysis of variance (ANOVA) test was done to assess differences in knowledge scores in relation to demographic characteristics. Statistical significance will be defined as a P value of less than 0.05, with a confidence interval of 95%. The statistical analysis was done by the Statistical Package for the Social Sciences (SPSS) software version 25 (IBM Corp., Armonk, NY).

Ethical considerations

This study was approved by the Bioethics Committee for Scientific and Medical Research at the University of Jeddah (approval no. UJ-REC-056). The consent was written on the first page of the questionnaire, stating that by submitting this questionnaire you agree to participate in the study. All information was kept confidential and only used for the purposes of scientific research.

## Results

Out of 392 participants, there were 146 in the age group of 19-25 (37.2%). Half of our participants were married (208; 53.1%). The majority were Saudis (336; 85.6%). More than half of the participants have a bachelor's degree (234; 59.7%). The participants’ mean knowledge score was 6.9 ± 3.36. Only 8% of the participants had a good level of knowledge while the remaining 92% had a poor level of knowledge (Table 1). The one-way ANOVA test demonstrated a significant statistical difference in mean knowledge scores across age groups (P=0.003), marital status (P=0.006), educational status (P=0.020), and occupations (P=0.001) (Table 2).

**Table 1:** Frequencies and percentage of knowledge level

Item	Frequency	Percentage
Poor knowledge	361	92.1
Good knowledge	31	7.9
Total	392	100

**Table 2:** Demographic characteristics and mean knowledge score by demographic variables for a sample of 392 participants‏

Demographics		Number of participants (%)	Knowledge score (mean ± standard deviation)	P-value
Age group (years)	19-25	146(37.2)	7.7±3.25	0.003
	26-35	90(23)	6.38±3.07	
	36-45	51(13)	6.5±3.28	
	46+	105(26.8)	6.39±3.6	
Nationality	Saudi	336(85.7)	6.89±3.4	0.922
	Non-Saudi	56(14.3)	6.9±2.9	
Marital status	Single	162(41.3)	7.56±3.38	0.006
	Married	208(53.1)	6.5±3.2	
	Divorced	13(3.3)	5.76±3.58	
	Widow	9(2.3)	5.3±4.1	
Education	Less than high school	11(2.8)	3.7±3.55	0.020
	High school	107(27.3)	7.2±3.36	
	Bachelor	234(59.7)	6.8±3.3	
	Master	27(6.9)	7.4±3.2	
	PhD	13(3.3)	7.3±2.89	
Occupation	Healthcare worker	38(9.7)	7.6±3.35	0.001
	Non-employee	193(49.2)	6.27±3.08	
	Employee	99(25.3)	6.3±3.28	
	Medical student	45(11.5)	9.5±3.07	
	Non-medical student	17(4.3)	8.8±3.7	
Do you have a family history of breast cancer of first-degree mother or sister	Yes	40(10.2)	7.5±3.5	0.220
	No	352(89.8)	6.8±3.3	
Knowledge score		392 (100)	6.9 ± 3.36	

Most of the participants are aware of breast cancer 372 (94.9%). Social media was the highest source of information regarding breast cancer 142 (37.8%) (Table 3).

**Table 3:** Breast cancer awareness among study participants (N=392)

Item	Response	Female	
Are you aware		N	%
	Yes	372	94.9%
	No	20	5.1%
Source of breast cancer information:	Social media	142	37.8%
	Relative-Friend	76	20.2%
	Medical staff	75	19.9%
	Television	31	8.3%
	Other	52	13.8%

The majority of the respondents reported that the main risk factor of breast cancer was family history (83.7%) followed by radiation exposure (50.0%), and old age and diet (30.9%). Nipple changes, nipple secretions, breast lumps, and changes in breast size and shape are the major symptoms of breast cancer (Table 4).

**Table 4:** Frequency and percentage of the scientific background of breast cancer

Risk factors of breast cancer		N	%
Family history	Yes	328	83.7
	No	64	16.3
Use of brassieres	Yes	68	17.3
	No	324	82.7
First child at late age	Yes	45	11.5
	No	347	88.5
Medical condition	Yes	115	29.3
	No	277	70.7
Diet	Yes	121	30.9
	No	271	69.1
Stress and anxiety	Yes	76	19.4
	No	316	80.6
Radiation exposure	Yes	196	50.0
	No	196	50.0
Intake of oral contraceptive	Yes	95	24.2
	No	297	75.8
No breastfeeding	Yes	89	22.7
	No	303	77.3
Old age	Yes	121	30.9
	No	271	69.1
Late menopause	Yes	60	15.3
	No	332	84.7
Excessive breastfeeding	Yes	9	2.3
	No	383	97.7
Not aware	Yes	14	3.6
	No	378	96.4
I do not know	Yes	37	9.4
	No	355	90.6
Symptoms of breast cancer			
Nipple changes	Yes	265	67.6
	No	127	32.4
Nipple secretions	Yes	230	58.7
	No	162	41.3
Breast lump	Yes	347	88.5
	No	45	11.5
Itching in the breast	Yes	81	20.7
	No	311	79.3
Change in breast size and shape	Yes	249	63.5
	No	143	36.5
Breast Pain and soreness	Yes	212	54.1
	No	180	45.9
Not aware	Yes	9	2.3
	No	383	97.7
I do not know	Yes	28	7.1
	No	364	92.9

**Figure 1:**
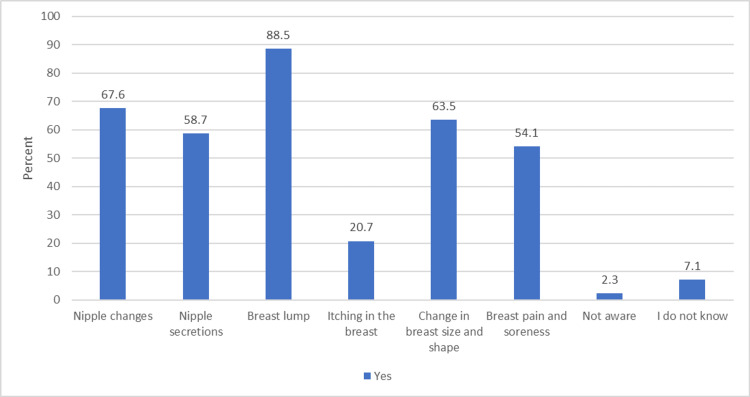
Frequency and percentage of symptoms of breast cancer

A breast lump was the most reported symptom of breast cancer (88.5%) followed by nipple changes (67.6%) and changes in breast size and shape (63.5%) (Figure 1).

Regarding knowledge of BSE, more than half of the respondents had heard about BSE but were without BSE practice (57.9%) because they had other reasons (46.0%). About 37% believe that the purpose of BSE practice is advice from a healthcare professional followed by a routine examination (37.3%). About 97% agreed that early detection of breast cancer increased the chance of recovery, and 82.1% agreed that females over 20 years should practice BSE frequently (Table 5).

**Table 5:** Knowledge and attitude of breast self-examination

		N	%
Knowledge of breast self-examination (BSE)	Never heard of BSE	47	12.0
	Heard about BSE but without BSE practice	227	57.9
	Heard of BSE and are practicing it	118	30.1
Why not practice BSE (n=227)	Too busy	77	34.1
	Not needed	30	13.3
	Inconvenient	15	6.6
	Others	105	46.0
Purpose of BSE practice (n=118)	Advice from a healthcare professional	44	37.3
	Noticed a breast lump	9	7.6
	Routine examination	44	37.3
	One of my relatives had cancer	16	13.6
	Medical reason	5	4.2
Attitude toward breast self-examination			
Early detection of breast cancer increases the chance of recovery	Agree	381	97.2
	Disagree	2	0.5
	Neutral	9	2.3
Females more than 20 years should practice BSE frequently	Agree	322	82.1
	Disagree	27	6.9
	Neutral	43	11.0
Females must be educated about BSE	Agree	373	95.2
	Disagree	8	2.0
	Neutral	11	2.8

## Discussion

Breast cancer is one of the most common cancers worldwide. Assessing females’ awareness of and attitude to breast cancer and BSE is of essential importance in early detection, which can decrease the mortality rate and improve the chances of recovery. In Saudi Arabia, the incidence of new cases and mortality has been growing in the past few years. In this study, we investigate the awareness of and attitude to the early detection of breast cancer and BSE [1].

We found that most of our respondents were married Saudi women with bachelor’s degrees and over the age of 26. Only one-tenth of those surveyed stated that they had a family history of breast cancer. Our study showed that Saudi women were aware of breast cancer. However, there is an inadequate understanding of other important aspects, including some breast cancer symptoms and some risk factors associated with the development of breast cancer. Unexpectedly, 92% of the participants had a poor level of knowledge although most of them had a high educational level (bachelor’s degree). The present study also confirmed that though most women have heard of breast self-examination, only a limited number perform it, and the reason was that they were too busy.

Our result regarding knowledge of breast cancer was consistent with the Alshahrani study in that most of the participants were aware, and social media was the most common source of information [12]. In the current sample, a large percentage disagreed that having a first child at a late age, late menopause, the use of oral contraceptives, not breastfeeding, medical condition, old age, and diet are considered risk factors for breast cancer, and these findings are similar to the Alrawili study [15]. These findings are contrary to the Alshahrani study, where the majority of the participants agreed that diet, not breastfeeding, and the use of oral contraceptives are risk factors for breast cancer [12]. According to the Elsayed study, predominantly the participants agreed that early menarche, late menopause, radiation exposure, and family history [16]. The three most recognized symptoms of breast cancer, as reported by our respondents, are breast lump, nipple changes, itching in the breast, and changes in breast size and shape. This is in line with two previous studies [11,13]. However, a large proportion of our sample didn’t recognize nipple secretions, breast pain, and soreness as symptoms of breast cancer.

Regarding BSE, the majority of our respondents stated that they heard about it but only one-third practiced it. Additionally, the main reasons for practicing BSE were either advice from a healthcare professional or routine examination. A similar pattern of results was obtained in the Alshahrani and Alrawili studies [12,15]. Surprisingly, these findings are contrary to a study conducted among female medical students, and the majority reported that they were not aware of BSE [4]. In general, the respondents showed a positive attitude toward BSE. Most of our participants agreed that early detection of breast cancer increases the chance of recovery and that females over 20 years of age should practice BSE regularly and must be educated about BSE. These results are similar to the Alshahrani study except that a relatively large percentage of participants disagreed that females more than 20 years should practice BSE frequently.

The different results of this study have important implications for breast cancer studies, probably due to the different healthcare systems, social classes, sample distribution, age groups, regions, and qualifications. Furthermore, these differences, particularly in the recognition of risk factors and symptoms, suggest the need for further research to better understand the factors that contribute to differences in breast cancer knowledge and attitudes among different populations in Saudi Arabia. Such research could inform the development of more effective and culturally appropriate breast cancer awareness and early detection programs.

The differences between the results of our study and others are likely due to variations in healthcare systems, social classes, sample distribution, age groups, regions, and educational levels. Moreover, differences in the recognition of risk factors and symptoms emphasize the need for further research to better understand the factors that contribute to variations in breast cancer knowledge and attitudes among different populations in Saudi Arabia.‏

This study has some limitations. The generalizability of the study's findings may be limited due to the non-probability sampling method and the use of a self-reported online questionnaire, which could lead to recall and social desirability biases.

## Conclusions

This study showed a lack of knowledge and awareness regarding the risk factors and symptoms of breast cancer. In addition, BSE is believed to be a useful tool in conservative societies such as Saudi Arabia. However, they showed a poor practice of BSE. On the other hand, there is an excellent attitude toward it. Social media and relatives/friends were the main sources of information regarding breast cancer. We recommend including BSE as a subject in schools and universities for females and implementing community health campaigns in shopping malls and various public gatherings to increase knowledge and practice, which will decrease breast cancer morbidity and mortality rates.
